# Association Between Treatment Sequencing and Overall Survival in Stage IV NSCLC With Brain Metastases: A National Cancer Database Study

**DOI:** 10.1111/1759-7714.70359

**Published:** 2026-07-19

**Authors:** Anand Shah, Pranav Gwalani, Merry Zhai, Ritik Goyal, Joshua Kra

**Affiliations:** ^1^ Department of Medicine Rutgers New Jersey Medical Newark New Jersey USA; ^2^ Department of Internal Medicine Icahn School of Medicine at Mount Sinai New York New York USA; ^3^ Division of Hematology/Oncology, Department of Medicine Rutgers New Jersey Medical School Newark New Jersey USA; ^4^ Rutgers Cancer Institute Newark New Jersey USA

## Abstract

**Background:**

The optimal sequencing of brain‐directed radiation and systemic therapy in stage IV non–small cell lung cancer (NSCLC) with brain metastases remains uncertain in the era of CNS‐active systemic agents. We evaluated survival outcomes using national real‐world data.

**Methods:**

We conducted a retrospective cohort study of adults diagnosed between 2010 and 2022 with stage IV NSCLC and brain metastases in the National Cancer Database who received both brain‐directed radiation and systemic therapy. Treatment sequence was classified as radiation‐first or systemic‐first based on initiation dates. Multivariable Cox proportional hazards models, stratified by treatment era (pre‐2015 vs. 2015+), assessed associations with overall survival (OS), adjusting for demographic, clinical, tumor, and treatment factors. Propensity score matching and delayed‐entry sensitivity analyses were performed to address confounding and immortal time bias.

**Results:**

Among 45 577 patients, 78.3% received radiation‐first and 21.7% received systemic therapy first. Unadjusted Kaplan–Meier analysis showed no significant difference in OS (log‐rank *p* = 0.624). In multivariable analysis, systemic‐first sequencing was associated with a modest increase in mortality (adjusted hazard ratio [aHR] 1.06; 95% CI: 1.04–1.09), which was consistent in propensity‐matched (HR 1.07; 95% CI: 1.04–1.11) and delayed‐entry analyses (aHR 1.09; 95% CI: 1.07–1.12). The use of systemic‐first therapy increased over time. Established prognostic factors demonstrated larger effect sizes.

**Conclusion:**

Systemic‐first sequencing was associated with a modest increase in adjusted mortality; however, the effect size was small relative to established prognostic factors and likely influenced by residual confounding and selection bias. These findings support individualized, multidisciplinary treatment decisions rather than a uniform sequencing strategy.

## Graphical abstract

Among 45 577 NCDB patients with stage IV NSCLC and brain metastases receiving brain‐directed radiation and systemic therapy, unadjusted OS was similar by initial sequence. Systemic‐first treatment showed a small adjusted mortality signal, likely influenced by residual confounding and selection bias, supporting individualized sequencing decisions.

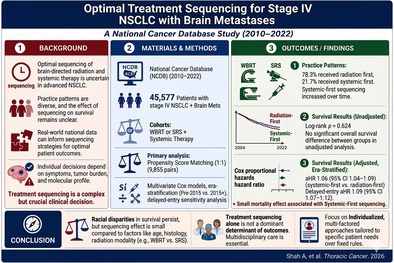

## 1 Introduction

Lung cancer is the leading cause of cancer‐related mortality worldwide and the most common primary malignancy to metastasize to the brain [1]. Approximately 10%–20% of patients with non‐small cell lung cancer (NSCLC) have brain metastases, and up to 50% develop intracranial disease during their illness [2]. Brain metastases pose substantial therapeutic challenges due to associated neurologic morbidity and historically limited central nervous system (CNS) penetration of systemic therapies.

Management has traditionally relied on brain‐directed radiation therapy, including whole‐brain radiotherapy (WBRT) or stereotactic radiosurgery (SRS), to achieve rapid intracranial control and reduce neurological symptoms [3]. This radiation‐first approach was reinforced by the limited CNS activity of conventional cytotoxic chemotherapy [4]. However, accumulating evidence indicates that selected chemotherapeutic agents have meaningful intracranial activity, challenging earlier assumptions of intrinsic CNS resistance [5, 6].

The therapeutic landscape has shifted with the advent of highly effective CNS‐penetrant systemic therapies. Targeted agents against oncogenic drivers (e.g., EGFR, ALK) demonstrate significant intracranial activity, often allowing deferred or omitted radiation in patients with asymptomatic disease [7, 8]. Immune checkpoint inhibitors (ICIs) have also shown intracranial responses in selected patients, further expanding first‐line systemic options [9]. Concurrently, SRS has increasingly replaced WBRT to better preserve neurocognition, aligning with a trend toward less morbid local interventions [10].

As a result of these advances, clinicians are increasingly faced with uncertainty regarding the optimal sequencing of brain‐directed radiation and systemic therapy in patients who are candidates for both modalities. In practice, sequencing is individualized, often prioritizing upfront radiation for symptomatic or bulky intracranial disease, while favoring initial systemic therapy for asymptomatic patients, particularly those with actionable oncogenic drivers [11, 12]. Existing evidence remains limited and largely context specific. Current multidisciplinary guidelines generally recommend upfront local therapy for symptomatic brain metastases, while allowing consideration of deferred radiation in selected asymptomatic patients when effective CNS‐active systemic therapies are available [13].

Prospective data are emerging in selected populations, including the phase II Atezo‐Brain study of atezolizumab‐based chemoimmunotherapy in advanced nonsquamous NSCLC with untreated brain metastases and the STARLET joint analysis of OUTRUN and LUOSICNS evaluating upfront SRS plus osimertinib versus osimertinib alone in EGFR‐mutant NSCLC, and the first phase III trial of upfront versus delayed cranial RT in oncogene‐mutated NSCLC with asymptomatic brain metastases [14, 15, 16]. Although prior molecularly defined cohorts have suggested potential benefit from initial SRS before targeted therapy, available evidence remains clinically or molecularly selected and does not fully address national practice patterns among patients receiving both brain‐directed radiation and systemic therapy [17].

Given these gaps, real‐world data may provide valuable insight into contemporary practice patterns and outcomes. We conducted a retrospective cohort study using the National Cancer Database (NCDB) to (1) evaluate national trends in treatment sequencing from 2010 to 2022, and (2) examine whether initial treatment sequence is associated with overall survival (OS) among patients with stage IV NSCLC and brain metastases who received both modalities.

## 2 Methods

### 2.1 Database

We conducted a retrospective cohort study using the NCDB, a hospital‐based oncology registry jointly sponsored by the American College of Surgeons Commission on Cancer and the American Cancer Society. The NCDB captures deidentified tumor, demographic, treatment, and outcome data from Commission on Cancer‐accredited facilities and includes approximately 72% of newly diagnosed cancers in the United States [18, 19]. Data are abstracted using standardized registry procedures and are released to investigators as participant use files under a data use agreement; the NCDB is not a publicly accessible database, and access requires an institutional application and executed data use agreement. Because the NCDB contains deidentified patient‐level data consistent with HIPAA Privacy Rule standards, individual patient consent was not required, and this study was exempt from institutional review board approval under 45 CFR 46.104(d)(4). This study followed the Strengthening the Reporting of Observational Studies in Epidemiology (STROBE) guidelines for cohort studies.

### 2.2 Participant Selection

Adult patients (age ≥ 18 years) diagnosed between 2010 and 2022 with stage IV NSCLC and documented brain metastases at presentation were identified. Patients who were untreated, had missing vital status or survival time, or underwent surgery as the initial treatment modality were excluded. Because the study question focused on sequencing of brain‐directed radiation therapy and systemic therapy, patients who received only radiation therapy, received only systemic therapy, or lacked known start dates for both modalities were excluded. Patients with same‐day initiation of radiation therapy and systemic therapy were also excluded to ensure unambiguous treatment sequencing. The final analytic cohort was restricted to patients receiving WBRT or SRS as the recorded brain‐directed radiation modality. Although the NCDB captures immunotherapy as a separate variable, it does not identify specific agents, and many targeted therapies, including EGFR and ALK inhibitors, are classified under chemotherapy; consequently, systemic therapy classes could not be reliably disaggregated.

### 2.3 Treatment Sequencing Definition

Treatment sequence was defined by recorded initiation dates. Patients were classified as radiation‐first if brain‐directed radiation preceded systemic therapy, and systemic‐first if systemic therapy preceded radiation. Patients with same‐day initiation of both modalities were excluded to ensure unambiguous classification. By restricting the cohort to patients who received both therapies, all included patients necessarily survived to receive multimodality treatment, thereby reducing immortal time bias.

### 2.4 Covariates

Covariates included age at diagnosis (categorized as < 65 vs. ≥ 65 years), sex, race/ethnicity, insurance status, Charlson–Deyo comorbidity index (CDCI), histology (adenocarcinoma, squamous cell, other/not otherwise specified), primary tumor site, facility type, urban–rural residence, radiation modality (SRS vs. WBRT), treatment era (pre‐2015 vs. 2015+), and time from diagnosis to treatment initiation of first therapy [20].

### 2.5 Statistical Analysis

Baseline characteristics were compared between treatment sequence groups using chi‐square tests for categorical variables and Wilcoxon rank‐sum tests for continuous variables. Overall survival (OS) was defined as time from diagnosis to death from any cause or last follow‐up. Survival was estimated using the Kaplan–Meier method and compared with log‐rank tests. Multivariable Cox proportional hazards regression, adjusting for all covariates listed above, was used to evaluate the association between treatment sequence and OS. The proportional hazards assumption was evaluated using Schoenfeld residuals. All survival analyses were stratified by treatment era (pre‐2015 vs. 2015+) to account for the introduction of immune checkpoint inhibitors for NSCLC management. A sensitivity analysis without era stratification was also performed.

### 2.6 Propensity Score Matching

To further address confounding by indication, propensity score matching (PSM) was performed. The propensity score was derived from multivariable logistic regression including all covariates listed above. Patients were matched 1:1 using nearest‐neighbor matching without replacement, with a caliper width of 0.01 on the propensity score scale. Covariate balance was assessed using standardized mean differences, with values < 10% considered well‐balanced. Within the matched cohort, OS was compared using Kaplan–Meier analysis with log‐rank testing, and the hazard ratio was estimated using unadjusted Cox proportional hazards regression.

### 2.7 Sensitivity and Temporal Analyses

To further address potential immortal time bias, a delayed‐entry sensitivity analysis was performed in which follow‐up began at initiation of the second treatment modality, ensuring that patients contributed person‐time only after receipt of both therapies. Cox proportional hazards regression with the same covariate adjustment and era stratification was applied. An interaction analysis between treatment sequence and radiation modality (SRS vs. non‐SRS) was performed. Temporal trends in treatment sequencing were assessed using logistic regression, with year of diagnosis modeled as a continuous variable.

## 3 Results

### 3.1 Baseline Characteristics

**FIGURE 1:**
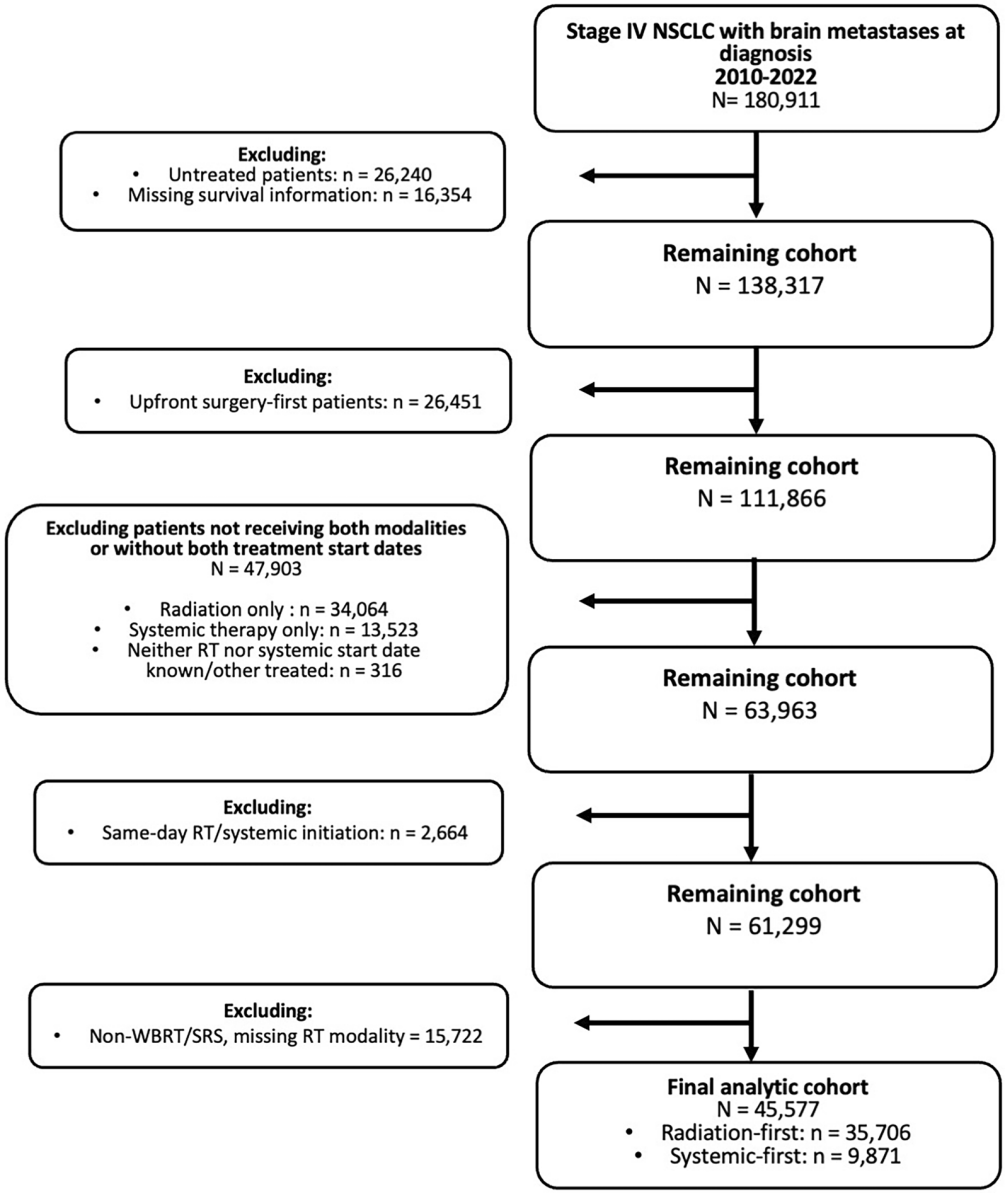
Patient selection flow diagram. Cohort derivation for patients with stage IV NSCLC and brain metastases at diagnosis in the NCDB from 2010 to 2022. After exclusions, the final analytic cohort included 45 577 patients: 35 706 radiation‐first and 9871 systemic‐first.

A total of 45 577 patients with stage IV NSCLC and brain metastases who received both brain‐directed radiation and systemic therapy between 2010 and 2022 were included (Figure 1). Of these, 35 706 patients (78.3%) received radiation therapy first, while 9871 patients (21.7%) received systemic therapy first.

Median OS for the cohort was 11.0 months (IQR, 4.8–24.2). Median survival was similar between groups: 10.5 months (IQR, 4.7–24.34) for the radiation‐first group and 10.6 months (IQR, 5.0–23.6) for the systemic‐first group.

Baseline characteristics stratified by treatment sequence are summarized in Table 1. Age distribution (median 64 years) and sex ratio were similar between groups. Patients receiving systemic therapy first were more frequently diagnosed in 2015 or later (72.3% vs. 69.1%), more likely to be treated at academic/research programs (37.0% vs. 33.7%), and had a longer median interval from diagnosis to treatment initiation (25 vs. 17 days). The systemic‐first group had a lower proportion of adenocarcinoma (73.4% vs. 79.1%) and a higher proportion of squamous cell (12.2% vs. 10.2%) and other/NOS histology (14.4% vs. 10.7%). Notably, patients in the systemic‐first group were substantially more likely to receive SRS rather than WBRT (43.0% vs. 32.4%). While statistically significant differences were observed across most covariates, absolute differences were generally small, consistent with the large sample size.

**TABLE 1:** Baseline demographic and clinical characteristics of patients with stage IV non–small cell lung cancer and brain metastases, stratified by initial treatment sequence.

Variables	Category	Overall (*n* = 45, 577)	Radiation‐first (*n* = 35,706)	Systemic‐first (*n* = 9871)	*p* value
Age	Median (IQR)	64 (57–71)	64 (57–71)	64 (57–71)	—
Age group	≤ 64	23,648 (51.9%)	18,682 (52.3%)	4966 (50.3%)	**< 0.001**
	≥ 65	21,929 (48.1%)	17,024 (47.7%)	4905 (49.7%)	
Sex	Male	22,221 (48.8%)	17,295 (48.4%)	4926 (49.9%)	**0.010**
	Female	23,356 (51.2%)	18,411 (51.6%)	4945 (50.1%)	
Race	White	35,017 (76.8%)	27,651 (77.4%)	7366 (74.6%)	**< 0.001**
	Black	5470 (12.0%)	4292 (12.0%)	1178 (11.9%)	
	Hispanic	1359 (3.0%)	1011 (2.8%)	348 (3.5%)	
	Asian	1979 (4.3%)	1403 (3.9%)	576 (5.8%)	
	Other/unknown	1752 (3.8%)	1349 (3.8%)	403 (4.1%)	
Facility type	Community cancer program	2929 (6.4%)	2348 (6.6%)	581 (5.9%)	**< 0.001**
	Comprehensive community program	17,185 (37.7%)	13,547 (37.9%)	3638 (36.9%)	
	Academic/research program	15,683 (34.4%)	12,036 (33.7%)	3647 (37.0%)	
	Integrated network cancer program	9265 (20.3%)	7405 (20.7%)	1860 (18.8%)	
	Unknown	515 (1.1%)	370 (1.0%)	145 (1.5%)	
Insurance status	Self‐pay	1635 (3.6%)	1352 (3.8%)	283 (2.9%)	**< 0.001**
	Private insurance	16,559 (36.3%)	12,968 (36.3%)	3591 (36.4%)	
	Medicaid	4976 (10.9%)	3924 (11.0%)	1052 (10.7%)	
	Medicare	21,058 (46.2%)	16,459 (46.1%)	4599 (46.6%)	
	Other/unknown	1349 (3.0%)	1003 (2.8%)	346 (3.5%)	
Urban–rural status	Metro	35,902 (78.8%)	28 027 (78.4%)	7875 (79.8%)	**< 0.001**
	Urban	5569 (12.2%)	4486 (12.6%)	1083 (11%)	
	Rural	1957 (4.3%)	1547 (4.3%)	410 (4.1%)	
	Not available	2149 (4.7%)	1646 (4.6%)	503 (5.1%)	
Charlson comorbidity index	0	31,450 (69.0%)	24,545 (68.7%)	6905 (70.0%)	**0.040**
	1	9347 (20.5%)	7348 (20.6%)	1999 (20.3%)	
	2	3016 (6.6%)	2416 (6.8%)	600 (6.1%)	
	≥ 3	1764 (3.9%)	1397 (3.9%)	367 (3.7%)	
Time from diagnosis to first treatment, days	Median (IQR)	19 (8–34)	17 (7–31)	25 (13–40)	**< 0.001**
Histology	Adenocarcinoma	35,496 (77.9%)	28,248 (79.1%)	7248 (73.4%)	**< 0.001**
	Squamous cell carcinoma	4857 (10.7%)	3657 (10.2%)	1200 (12.2%)	
	Other/NSCLC NOS	5224 (11.5%)	3801 (10.7%)	1423 (14.4%)	
Primary site	Upper lobe	24,116 (52.9%)	19,160 (53.7%)	4956 (50.2%)	**< 0.001**
	Middle lobe	1793 (3.9%)	1400 (3.9%)	393 (4.0%)	
	Lower lobe	11,684 (25.6%)	9203 (25.8%)	2481 (25.1%)	
	Main bronchus	1587 (3.5%)	1188 (3.3%)	399 (4.0%)	
	Other/overlapping/NOS	6397 (14.0%)	4755 (13.3%)	1642 (16.6%)	
Era of diagnosis	Pre‐2015	13,773 (30.2%)	11,038 (30.9%)	2735 (27.7%)	**< 0.001**
	2015+	31,804 (69.8%)	24,668 (69.1%)	7136 (72.3%)	
Radiation modality	WBRT	29,779 (65.3%)	24,149 (67.6%)	5630 (57.0%)	**< 0.001**
	SRS	15,798 (34.7%)	11,557 (32.4%)	4241 (43.0%)	

*Note:* Data are presented as *n*(%) unless otherwise specified. *p* values were calculated using *χ* ^2^ tests for categorical variables and the Wilcoxon rank‐sum test for continuous variables. Given the large sample size, small distributional differences may achieve statistical significance without clinical relevance; therefore, emphasis is placed on absolute differences rather than *p* values. Bold values indicate statistical significance (*P* < 0.05).

Abbreviations: IQR, interquartile range; NOS, not otherwise specified.

### 3.2 Multivariate Survival Analysis

Results of the multivariable Cox proportional hazards model for overall survival are presented in Table 2. After adjustment for demographic, clinical, tumor, and treatment factors with stratification by era, systemic‐first sequencing was associated with a modest increase in mortality compared with radiation‐first sequencing (adjusted hazard ratio [aHR], 1.06; 95% CI: 1.04–1.09; *p* < 0.001).

**TABLE 2:** Multivariable Cox proportional hazards model for overall survival in patients with stage IV NSCLC and brain metastases.

Variables	Adjusted hazard ratio (95% CI)	*p* value
Treatment sequence (ref: Radiation first)		
Systemic first	1.06 (1.04–1.09)	**< 0.001**
Age group (ref: < 65years)		
≥ 65 years	1.27 (1.24–1.31)	**< 0.001**
Sex (ref: male)		
Female	0.84 (0.82–0.86)	**< 0.001**
Race/ethnicity (ref: White)		
Hispanic	0.85 (0.80–0.91)	**< 0.001**
Black	0.93 (0.90–0.96)	**< 0.001**
Asian	0.70 (0.67–0.74)	**< 0.001**
Other/unknown	0.96 (0.91–1.01)	0.142
Insurance status (ref: Private insurance)		
Self‐pay	1.14 (1.08–1.21)	**< 0.001**
Medicaid	1.15 (1.11–1.20)	**< 0.001**
Medicare	1.10 (1.07–1.14)	**< 0.001**
Other/unknown	1.03 (0.97–1.10)	0.173
Charlson‐Deyo comorbidity index (ref: 0)		
1	1.09 (1.06–1.11)	**< 0.001**
2	1.19 (1.14–1.24)	**< 0.001**
≥ 3	1.26 (1.20–1.33)	**< 0.001**
Histology (ref: Adenocarcinoma)		
Squamous cell carcinoma	1.44 (1.39–1.49)	**< 0.001**
Other/NSCLC NOS	1.30 (1.26–1.34)	**< 0.001**
Primary tumor site (ref: Upper lobe)		
Middle lobe	1.01 (0.96–1.06)	0.807
Lower lobe	1.04 (1.01–1.06)	**0.002**
Main bronchus	1.12 (1.06–1.18)	**< 0.001**
Other/NOS	1.11 (1.08–1.14)	**< 0.001**
Facility type (ref: Community cancer program)		
Comprehensive community program	0.95 (0.91–0.99)	**0.01**
Academic/research program	0.83 (0.79–0.86)	**< 0.001**
Integrated network cancer program	0.92 (0.88–0.96)	**< 0.001**
Urban–rural residence (ref: Metro)		
Urban	1.04 (1.01–1.07)	**0.017**
Rural	1.10 (1.05–1.16)	**< 0.001**
Radiation modality (ref: WBRT)		
SRS	0.74 (0.72–0.76)	**< 0.001**
Time from diagnosis to first treatment initiation (continuous) Per day increase	0.994 (0.994–0.995)	**< 0.001**

*Note:* Models were stratified by treatment era (pre‐2015 vs. 2015 and later). Bold values indicate statisitcal significance (*P* < 0.05).

Abbreviations: CI, confidence interval; NOS, not otherwise specified.

Several patient‐ and tumor‐related factors showed substantially larger associations with mortality. Age of ≥ 65 years was associated with a higher hazard of death (aHR, 1.27; 95% CI: 1.24–1.31). Increasing comorbidity burden was associated with progressively worse survival (CDCI score 1: aHR, 1.09; 95% CI: 1.06–1.12; CDCI 2: aHR, 1.19; 95% CI: 1.14–1.24; CDCI ≥ 3: aHR, 1.27; 95% CI: 1.20–1.33; all *p* < 0.001). Female sex was associated with improved survival (aHR, 0.84; 95% CI: 0.82–0.86).

Compared with White patients, Hispanic (aHR, 0.85; 95% CI: 0.80–0.91), Black (aHR, 0.93; 95% CI: 0.90–0.96), and Asian patients (aHR, 0.70; 95% CI: 0.67–0.74) had lower hazards of mortality. Relative to private insurance, uninsured (aHR, 1.14; 95% CI: 1.08–1.21), Medicaid (aHR, 1.15; 95% CI: 1.11–1.19), and Medicare (aHR, 1.11; 95% CI: 1.07–1.14) were associated with worse survival.

Squamous cell carcinoma (aHR, 1.44; 95% CI: 1.40–1.49) and NSCLC not otherwise specified (aHR, 1.30; 95% CI: 1.26–1.34) were associated with higher mortality compared with adenocarcinoma. Treatment at academic/research programs was associated with improved survival compared with community cancer programs (aHR, 0.83; 95% CI: 0.79–0.86). Receipt of SRS compared with WBRT was associated with substantially lower mortality (aHR, 0.74; 95% CI: 0.72–0.76).

In sensitivity analyses without stratification by treatment era, systemic‐first sequencing was associated with increased mortality compared to radiation‐first sequencing (aHR 1.05; 95% CI: 1.03–1.08) (Table S1).

### 3.3 Kaplan–Meier Survival Analysis

**FIGURE 2:**
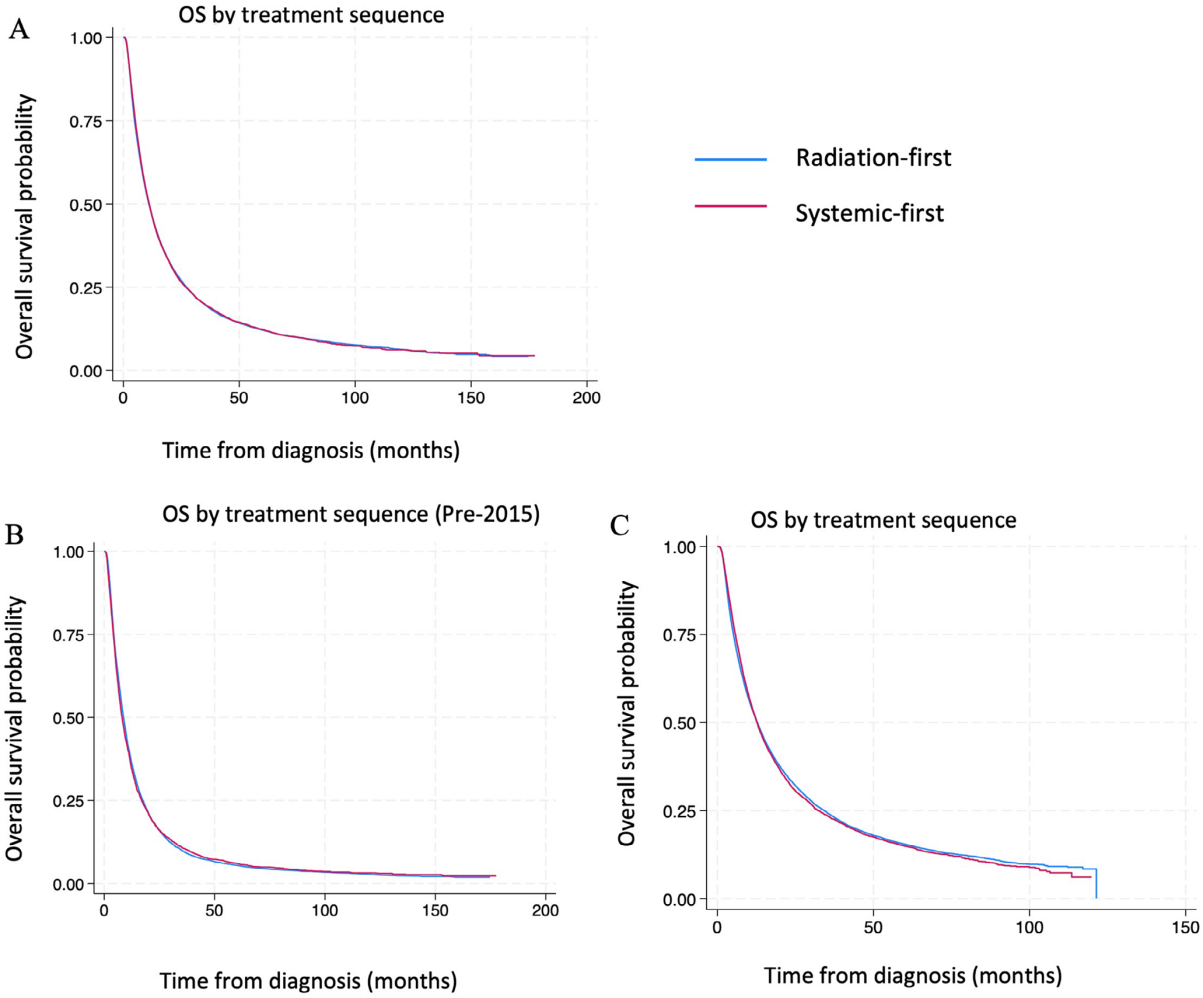
Kaplan–Meier curves for overall survival by treatment sequence in patients with stage IV NSCLC and brain metastases. (A) Overall cohort (log‐rank *p* = 0.624). (B) Patients diagnosed in the pre‐2015 era (log‐rank *p* = 0.421). (C) Patients diagnosed in the 2015 and later era (log‐rank *p* = 0.756). Survival was measured from date of diagnosis. No statistically significant difference in unadjusted overall survival was observed between radiation‐first and systemic‐first sequencing in the overall cohort or within either treatment era.

In unadjusted Kaplan–Meier analysis, no statistically significant difference in overall survival was observed between radiation‐first and systemic‐first strategies in the overall cohort (log‐rank *p* = 0.624; Figure 2A). When stratified by era, no difference was observed among patients diagnosed prior to 2015 (log‐rank *p* = 0.421; Figure 2B) or in 2015 and later (log‐rank *p* = 0.756; Figure 2C). Despite the absence of unadjusted survival differences, multivariable Cox regression identified a modest increase in adjusted mortality associated with systemic‐first sequencing (aHR, 1.06; 95% CI: 1.04–1.09).

### 3.4 Sensitivity and Exploratory Analyses

In a delayed‐entry sensitivity analysis with follow‐up beginning at receipt of the second treatment modality, systemic‐first sequencing remained associated with increased mortality (aHR, 1.09; 95% CI: 1.07–1.12; *p* < 0.001; Table S2). Associations for other covariates were consistent with the primary model. In an interaction analysis, no significant effect modification by radiation modality was observed (*p* = 0.115). The association between systemic‐first sequencing and mortality was consistent across radiation subgroups, with point estimates suggesting modestly increased mortality in both WBRT‐treated (aHR, 1.05; 95% CI: 1.01–1.08) and SRS‐treated patients (aHR, 1.09; 95% CI: 1.05–1.13).

### 3.5 Propensity Score Matching

A total of 9855 systemic‐first patients were successfully matched to 9855 radiation‐first patients (19 710 total), with 10 systemic‐first patients falling outside the region of common support and being excluded. After matching, covariate balance improved substantially across all variables. Mean standardized bias decreased from 8.4% to 1.9%, and the pseudo‐*R* ^2^ of the propensity score model decreased from 0.024 to 0.001, indicating successful balance (Table 3; Figure S1). Histology, which had the largest pre‐match imbalance (13.7% standardized difference), was reduced to 0.2% after matching. Time from diagnosis to treatment initiation showed the greatest residual imbalance (3.9% standardized difference) but remained below conventional thresholds.

**TABLE 3:** Covariate balance before and after propensity score matching.

Variables	Unmatched standardized bias (%)	Matched standardized bias (%)	% bias reduction
Age ≥ 65 years	4.1	3	26.9
Sex: female versus male	3	0.8	74.1
Race/ethnicity	5.1	3	41
Insurance status	3.5	2.6	26.4
Charlson‐Deyo index	2.9	2.2	23.2
Histology	13.7	0.2	98.8
Primary tumor site	9.6	0.9	90.4
Treatment era (2015+)	7	0.8	88
Days from diagnosis to treatment	26.6	3.9	85.4
Facility type	2.9	0.3	88.8
Urban–rural residence	1.1	1.6	−43.0
Radiation modality (SRS)	22	4	81.6
Overall mean bias	8.4	1.9	77.4
Pseudo‐*R* ^2^	0.024	0.001	—

*Note:* Standardized bias represents the standardized mean difference between treatment‐sequence groups before and after propensity score matching. “Sex: female versus male” and “Radiation modality: SRS versus WBRT” are binary contrasts. For multi‐level categorical variables, the reported value summarizes overall covariate imbalance across categories. Values < 10% were considered well balanced.

**FIGURE 3:**
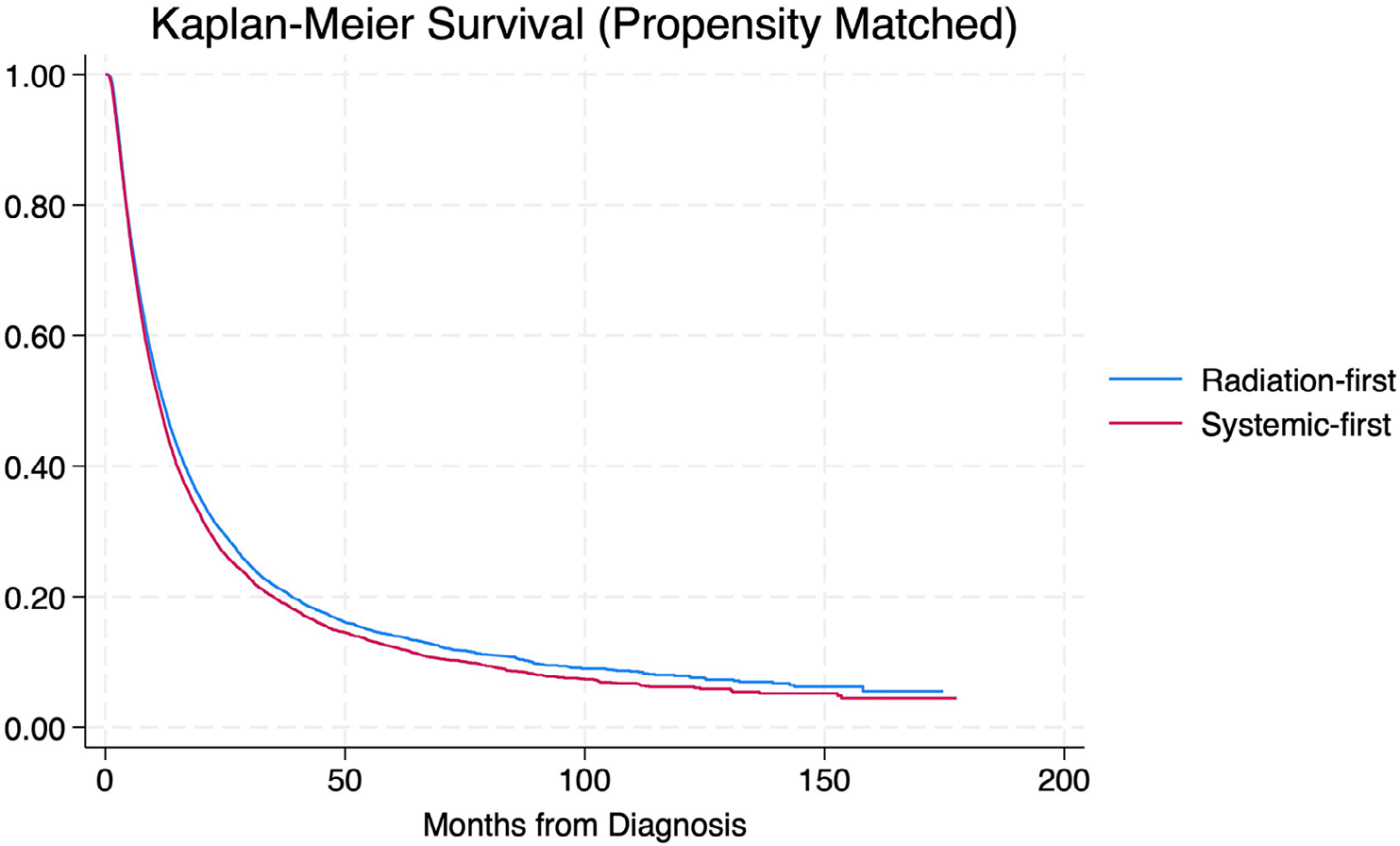
Kaplan–Meier survival curves in the propensity score–matched cohort. Overall survival by treatment sequence among 19 710 propensity score–matched patients (9855 per group). Systemic‐first sequencing was associated with significantly worse survival (log‐rank *p* < 0.001; HR 1.07; 95% CI: 1.04–1.11).

In the propensity score–matched cohort, systemic‐first sequencing was associated with worse overall survival compared with radiation‐first sequencing (log‐rank *p* < 0.001) (Figure 3). Cox regression in the matched cohort demonstrated a 7% increase in the hazard of death with systemic‐first sequencing (HR 1.07; 95% CI: 1.04–1.11; *p* < 0.001).

### 3.6 Temporal Trends in Treatment Sequencing

**FIGURE 4:**
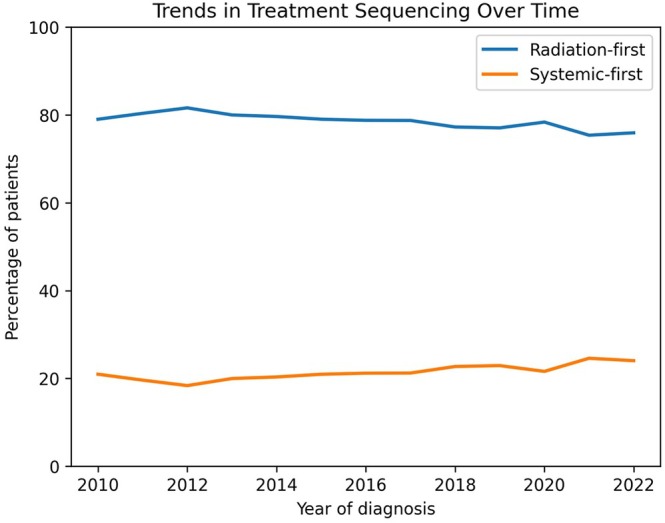
Trends in treatment sequencing among stage IV NSCLC with brain metastases from 2010 to 2022. The proportion of patients treated with radiation first decreased slightly over time, while the proportion treated with systemic therapy first increased.

Over the study period, the proportion of patients receiving systemic therapy first increased steadily, while the use of radiation‐first sequencing declined (Figure 4). In adjusted logistic regression analyses, the likelihood of receiving systemic therapy first increased significantly over time (adjusted odds ratio per year, 1.03; 95% CI: 1.02–1.04; *p* < 0.001). This temporal trend persisted after adjustment for demographic, clinical, and tumor‐related characteristics.

## 4 Discussion

In this national cohort of 45 577 patients with stage IV NSCLC and brain metastases who received both brain‐directed radiation and systemic therapy, systemic‐first sequencing was consistently associated with modestly worse survival across multiple analytic approaches. The primary multivariable analysis demonstrated a 6% increase in adjusted mortality (aHR 1.06; 95% CI: 1.04–1.09); propensity score matching yielded a 7% increase (HR 1.07; 95% CI: 1.04–1.11); and delayed‐entry analysis showed a 9% increase (aHR 1.09; 95% CI: 1.07–1.12). Unadjusted Kaplan–Meier analyses demonstrated no significant survival difference overall or within either treatment era, while the propensity‐matched comparison reached significance (log‐rank *p* < 0.001), suggesting that confounding by indication partially obscured the sequencing effect in the unmatched cohort.

The consistent 6%–9% increase in adjusted mortality across multivariable, propensity‐matched, and delayed‐entry analyses suggests a real but modest association between systemic‐first sequencing and worse survival. However, the clinical significance of this effect size warrants careful interpretation. The magnitude is small relative to established prognostic factors in this cohort: age ≥ 65 years (aHR 1.27), squamous histology (aHR 1.44), and Medicaid or uninsured status (aHR 1.14–1.15), all of which carried substantially larger hazards. These findings provide population‐level reassurance that the evolving practice of initiating CNS‐active systemic therapy, increasingly supported by data in molecularly selected patients, does not confer a major survival disadvantage [21, 22, 23]. The significant temporal shift toward systemic‐first sequencing, alongside increased use of stereotactic techniques, reflects the integration of more effective therapies into routine care [5, 24]. These data support individualized, multidisciplinary sequencing decisions tailored to neurological symptom burden, intracranial disease volume, molecular profile, and performance status, rather than a rigid radiation‐first algorithm [23, 25]. This interpretation is consistent with emerging prospective data, including Atezo‐Brain and STARLET, which highlight that sequencing decisions are likely dependent on systemic therapy class, molecular subtype, neurologic symptoms, and intracranial disease burden. Because these variables are not captured with sufficient granularity in the NCDB, our findings should be viewed as population‐level real‐world context that complements, rather than replaces, prospective molecularly defined studies [14, 15].

Despite the consistency of the adjusted signal across analytic approaches, residual confounding likely contributes to the observed association. Unadjusted Kaplan–Meier analyses showed no survival difference by treatment sequence, and the presence of a modest hazard only after covariate adjustment is characteristic of confounding by indication. Patients selected for systemic‐first therapy likely differ systematically from those treated with upfront radiation with respect to intracranial disease burden, neurologic symptoms, performance status, and molecular profile, factors not captured in the NCDB. Propensity score matching improved balance on measured covariates but cannot account for these unmeasured clinical factors, which are central to real‐world sequencing decisions. In exploratory interaction analysis, the systemic‐first hazard appeared larger among patients treated with SRS (aHR 1.09; 95% CI: 1.05–1.13) than among those receiving WBRT (aHR 1.05; 95% CI: 1.01–1.08), though the interaction term did not reach statistical significance (*p* = 0.115). This pattern may reflect differences in patient selection; patients receiving SRS typically have limited intracranial disease amenable to focal treatment, and the decision to initiate systemic therapy first in this subgroup may identify patients with competing extracranial disease burden or other unmeasured risk factors rather than a direct adverse effect of sequencing itself.

Beyond survival, toxicity and tolerability are critical considerations that may influence sequencing choices. Combined modality therapy, particularly immunotherapy or targeted agents with radiotherapy, can increase the risk of adverse events such as radionecrosis [26]. For vulnerable populations, including the elderly or those with poor performance status, sequencing flexibility may be important to maximize tolerability and completion [27, 28]. Our finding of no major survival difference supports the clinician's ability to make such toxicity‐informed decisions.

Our analysis identified and confirmed several well‐established predictors of poor survival [29, 30, 31]. These included older age, higher comorbidity burden, SCC histology, and Medicaid or uninsured status. Receipt of SRS was associated with markedly lower mortality compared with WBRT (aHR 0.74), though this likely reflects selection bias, as patients receiving SRS typically have more favorable intracranial disease characteristics. Treatment at academic/research programs was also associated with improved survival (aHR 0.83), consistent with prior NCDB analyses demonstrating facility‐level outcome differences. We also observed lower mortality among female patients and among Hispanic, Black, and Asian patients compared to White patients. These findings likely reflect differential distribution of actionable driver mutations, which are more prevalent in never‐smokers, women, and Asian patients, as well as unmeasured differences in social determinants of health and treatment patterns, rather than inherent biological effects of race or sex [32].

Several limitations merit consideration. First, restricting the analytic cohort to patients who received both brain‐directed radiation therapy and systemic therapy introduces potential selection bias. Patients who received radiation therapy alone may have had greater neurologic symptom burden, higher intracranial disease burden, poor performance status, rapid clinical decline, or early mortality before systemic therapy could be initiated. Conversely, patients treated with systemic therapy alone may have had asymptomatic or limited intracranial disease, targetable molecular alterations, or clinical features supporting deferral of radiation. Therefore, our findings apply to patients selected to receive both modalities and should not be generalized to all patients with stage IV NSCLC and brain metastases. As an administrative database, the NCDB lacks granular clinical details such as performance status, neurologic symptom severity, number and size of brain metastases, corticosteroid use, molecular subtype, and reasons for treatment selection. Critically, it does not reliably distinguish between classes of systemic therapy, such as immunotherapy versus targeted therapy. These unmeasured factors are central to sequencing decisions and represent key sources of residual confounding. The NCDB also does not capture cause‐specific mortality, intracranial progression, neurocognitive outcomes, or treatment‐related toxicity, all of which are clinically relevant endpoints in this population. Nonetheless, the large sample size, contemporary timeframe, and use of multiple analytic approaches to address bias strengthen the robustness of our findings.

In summary, among patients with stage IV NSCLC and brain metastases who received both brain‐directed radiation and systemic therapy, treatment sequencing was associated with a modest difference in overall survival, with a slightly higher adjusted mortality risk for systemic‐first sequencing. However, the effect size was small relative to established prognostic factors and was likely influenced by residual confounding and selection bias. These findings suggest that treatment sequence alone is not a dominant determinant of outcomes and support individualized sequencing decisions based on clinical context, including symptom burden, intracranial disease volume, and molecular profile. Prospective studies and registries incorporating molecular subtype, intracranial response, and patient‐reported outcomes will be essential to define optimal sequencing strategies for specific clinical scenarios.

## Author Contributions

**Pranav Gwalani:** methodology, formal analysis. **Ritik Goyal:** writing – review and editing, writing – original draft. **Merry Zhai:** writing – review and editing, writing – original draft. **Joshua Kra:** writing – original draft, writing – review and editing, conceptualization, supervision. **Anand Shah:** conceptualization, methodology, formal analysis, project administration, writing – review and editing, writing – original draft.

## Funding

The authors have nothing to report.

## Conflicts of Interest

The authors declare no conflicts of interest.

## Supporting information


**Table S1:** Multivariable Cox proportional hazards model for overall survival without era stratification.
**Table S2:** Delayed‐entry Cox proportional hazards model for overall survival with follow‐up beginning at receipt of second treatment modality.
**Figure S1:** Standardized bias before and after propensity score matching. Standardized percent bias across covariates before and after 1:1 propensity score matching. Circles represent unmatched covariate imbalance and crosses represent matched covariate imbalance. Values closer to zero indicate improved balance after matching.

## Data Availability

The data that support the findings of this study are available from the American College of Surgeons National Cancer Database (NCDB) Participant Use File. Restrictions apply to the availability of these data, which were used under license for this study and are therefore not publicly available from the authors. Data may be obtained directly from the NCDB through its application process for eligible investigators.
