# Supplementary material for: Association Between Treatment Sequencing and Overall Survival in Stage IV NSCLC With Brain Metastases: A National Cancer Database Study
Source: Thorac Cancer. 2026 Jul 19;17(14):e70359. doi: 10.1111/1759-7714.70359 (PMC13381076; doi:10.1111/1759-7714.70359)

**Supplementary Table S1. Multivariable cox proportional hazards model for overall survival without era stratification**

| Variable | Adjusted Hazard Ratio (95% CI) | P value |
| --- | --- | --- |
| **Treatment sequence (ref: Radiation first)** |  |  |
| Systemic first | 1.05 (1.03–1.08) | **<0.001** |
| **Age group (ref: ≤64 years)** |  |  |
| ≥65 years | 1.25 (1.21–1.29) | **<0.001** |
| **Sex (ref: Male)** |  |  |
| Female | 0.84 (0.83–0.86) | **<0.001** |
| **Race/Ethnicity (ref: White)** |  |  |
| Hispanic | 0.84 (0.79–0.89) | **<0.001** |
| Black | 0.92 (0.89–0.95) | **<0.001** |
| Asian | 0.70 (0.66–0.74) | **<0.001** |
| Other/Unknown | 0.97 (0.92–1.02) | 0.205 |
| **Insurance status (ref: Private insurance)** |  |  |
| Uninsured | 1.17 (1.11–1.24) | **<0.001** |
| Medicaid | 1.12 (1.08–1.16) | **<0.001** |
| Medicare | 1.09 (1.06–1.13) | **<0.001** |
| Other/Unknown | 1.02 (0.96–1.08) | 0.611 |
| **Charlson-Deyo comorbidity index (ref: 0)** |  |  |
| 1 | 1.10 (1.07–1.12) | **<0.001** |
| 2 | 1.18 (1.13–1.22) | **<0.001** |
| ≥3 | 1.21 (1.15–1.28) | **<0.001** |
| **Histology (ref: Adenocarcinoma)** |  |  |
| Squamous cell carcinoma | 1.46 (1.41–1.51) | **<0.001** |
| Other/NSCLC NOS | 1.38 (1.34–1.43) | **<0.001** |
| **Primary tumor site (ref: Upper lobe)** |  |  |
| Middle lobe | 1.01 (0.96–1.06) | 0.741 |
| Lower lobe | 1.04 (1.01–1.06) | **0.005** |
| Main bronchus | 1.11 (1.05–1.17) | **<0.001** |
| Other/NOS | 1.11 (1.08–1.14) | **<0.001** |
| **Facility type (ref: Community Cancer Program)** |  |  |
| Comprehensive Community Program | 0.94 (0.90–0.98) | **0.002** |
| Academic/Research Program | 0.82 (0.78–0.85) | **<0.001** |
| Integrated Network Cancer Program | 0.91 (0.87–0.95) | **<0.001** |
| **Urban-rural residence (ref: Metro)** |  |  |
| Urban | 1.03 (1.00–1.07) | **0.043** |
| Rural | 1.09 (1.04–1.15) | **0.001** |
| **Radiation modality (ref: WBRT)** |  |  |
| SRS | 0.71 (0.70–0.73) | **<0.001** |
| Time from diagnosis to treatment (per day) | 0.995 (0.995–0.995) | **<0.001** |

Abbreviations: NOS, not otherwise specified; CI, confidence interval; WBRT, whole-brain radiotherapy; SRS, stereotactic radiosurgery. Model was not stratified by treatment era.

**Supplementary Table S2. Delayed-Entry Cox proportional hazards model for Overall Survival with follow-up beginning at receipt of second treatment modality**

| Variable | Adjusted Hazard Ratio (95% CI) | P value |
| --- | --- | --- |
| **Treatment sequence (ref: Radiation first)** |  |  |
| Systemic first | 1.09 (1.07–1.12) | **<0.001** |
| **Age group (ref: ≤64 years)** |  |  |
| ≥65 years | 1.26 (1.23–1.30) | **<0.001** |
| **Sex (ref: Male)** |  |  |
| Female | 0.84 (0.82–0.86) | **<0.001** |
| **Race/Ethnicity (ref: White)** |  |  |
| Hispanic | 0.86 (0.80–0.91) | **<0.001** |
| Black | 0.94 (0.91–0.97) | **<0.001** |
| Asian | 0.70 (0.66–0.74) | **<0.001** |
| Other/Unknown | 0.96 (0.91–1.01) | 0.132 |
| **Insurance status (ref: Private insurance)** |  |  |
| Uninsured | 1.15 (1.09–1.22) | **<0.001** |
| Medicaid | 1.16 (1.12–1.21) | **<0.001** |
| Medicare | 1.11 (1.08–1.15) | **<0.001** |
| Other/Unknown | 1.04 (0.98–1.11) | 0.203 |
| **Charlson-Deyo comorbidity index (ref: 0)** |  |  |
| 1 | 1.09 (1.06–1.12) | **<0.001** |
| 2 | 1.20 (1.15–1.25) | **<0.001** |
| ≥3 | 1.27 (1.21–1.34) | **<0.001** |
| **Histology (ref: Adenocarcinoma)** |  |  |
| Squamous cell carcinoma | 1.44 (1.40–1.49) | **<0.001** |
| Other/NSCLC NOS | 1.31 (1.27–1.35) | **<0.001** |
| **Primary tumor site (ref: Upper lobe)** |  |  |
| Middle lobe | 1.00 (0.95–1.06) | 0.86 |
| Lower lobe | 1.04 (1.01–1.06) | **0.002** |
| Main bronchus | 1.12 (1.06–1.18) | **<0.001** |
| Other/NOS | 1.11 (1.08–1.14) | **<0.001** |
| **Facility type (ref: Community Cancer Program)** |  |  |
| Comprehensive Community Program | 0.94 (0.90–0.98) | **0.004** |
| Academic/Research Program | 0.83 (0.79–0.87) | **<0.001** |
| Integrated Network Cancer Program | 0.92 (0.87–0.96) | **<0.001** |
| **Urban-rural residence (ref: Metro)** |  |  |
| Urban | 1.04 (1.00–1.07) | **0.026** |
| Rural | 1.09 (1.04–1.15) | **<0.001** |
| **Radiation modality (ref: WBRT)** |  |  |
| SRS | 0.73 (0.72–0.75) | **<0.001** |
| **Time from diagnosis to treatment (per day)** |  |  |
| Per day increase | 0.999 (0.998–0.999) | **<0.001** |

Footnote: Models were stratified by treatment era (pre-2015 vs 2015 and later). Follow-up time began at initiation of the second treatment modality to address immortal time bias.


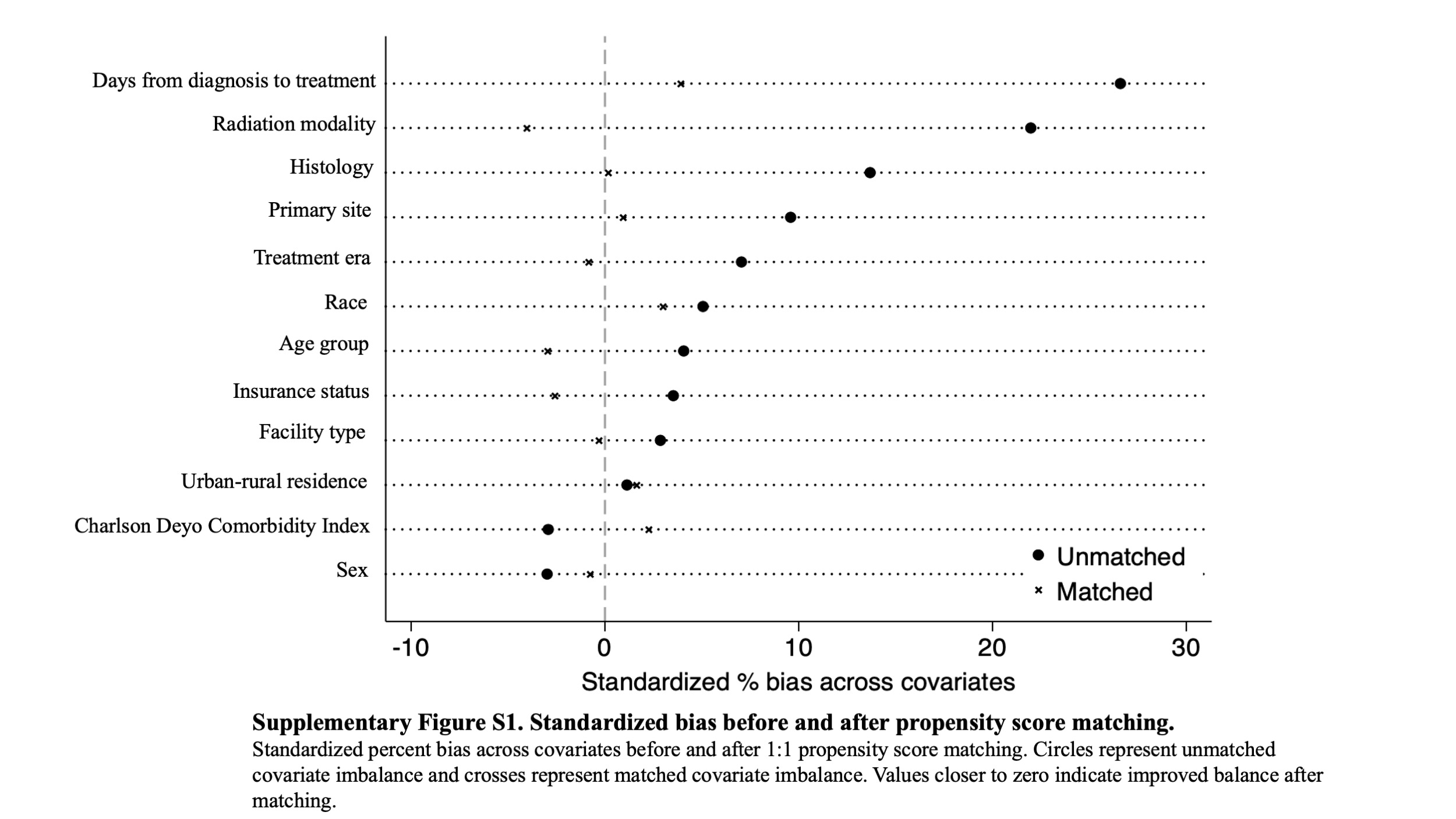

Supplement: Supplementary file 1 — Table S1: Multivariable Cox proportional hazards model for overall survival without era stratification. Table S2: Delayed‐entry Cox proportional hazards model for overall survival with follow‐up beginning at receipt of second treatment modality. Figure S1: Standardized bias before and after propensity score matching. Standardized percent bias across covariates before and after 1:1 propensity score matching. Circles represent unmatched covariate imbalance and crosses represent matched covariate imbalance. Values closer to zero indicate improved balance after matching. [file TCA-17-e70359-s001.docx]
